# Reflection of strengthening results in values of generalized degrees of metallicity and covalence is principle to new strategy of designing alloys

**DOI:** 10.1038/s41598-020-58560-z

**Published:** 2020-02-06

**Authors:** Evgeny Protopopov, Syuzanna Dobrykh, Yulia Trofimova, Pavel Malenko, Alexander Valter, Alexander Protopopov

**Affiliations:** Tula State University, Tula, prospect Lenina 92, 300012 Russia

**Keywords:** Condensed-matter physics, Materials science

## Abstract

To the best of our knowledge, the general approach of designing alloys with specified mechanical properties does not exist. This is due to the unresolved problem of analysing the set of heterogeneous variables that affect the mechanical properties along its production line from the smelting of the alloy to the manufacture of the final product. Here, we show that in principle this problem can be solved by analysing all the strengthening mechanisms in a common reference frame with reference to the single factor namely, the generalized degree of metallicity and covalence, which characterizes the entire interatomic bonds in all phases of the alloy. Such factors are able to reflect the results of hardening by various mechanisms because of the correlation with the mechanical properties. From the energy view point, these factors correspond to the proportion of the metallic and covalent bonds energy in the total energy of all chemical bonds in the alloy. Based on the approach being developed, we considered a method for predicting new doping systems for dispersively strengthening aluminum alloys according to the criterion of a given strength and have considered the methodology of optimizing chemical composition in steel smelting which is used for mass production of parts according to the criterion of the desired mechanical properties obtained due to solid solution hardening.

## Introduction

The search for new materials with desired properties is necessary for the long-term success of the technological sector^1^. A number of private approaches are used for the designing of new alloys. For example, this approach is an analysis of the electronic structure according to the electron density functional theory in the case when the exchange correlation potential is known^2^; it involves using regression dependencies^3^, or various semi-empirical models and the application of ab initio methods^4–6^. To the best of our knowledge, the general approach of designing alloys with specified mechanical properties does not exist. This is due to the unresolved problem of analysing the set of heterogeneous variables that affect the mechanical properties along its production line from the smelting of the alloy to the manufacture of the final product^7,8^, and also factors reflecting the interaction of structural, chemical and microstructural degrees of freedom^3^.

A structure and phase composition are usually characterized descriptively on the basis of visual observation or instrumental investigations or can be analyzed using various empirically obtained diagrams for example diagram of states including isothermal and thermokinetic diagrams, etc. in metallography. Point, linear, planar and bulk defects are considered using various mathematical apparatus. The factors of technological processes from alloy smelting to obtaining a finished product are usually described from the perspective of ensuring the possibility of optimal control. To create a common approach to the designing of various structural alloys, it is necessary with common positions to consider the totality of factors and variables listed above that determine the mechanical properties. Here we show that in principle this problem can be solved by analysing all the strengthening mechanisms in a common reference frame with regards to the single factors, namely generalized degrees of metallicity and covalence, which characterizes the total interatomic bonds in all phases of the alloy. Such factors are able to reflect the results of hardening by various mechanisms because of the correlation with the mechanical properties.

According to W. Harrison^9^, the degrees of metallicity and covalence characterize the energies of metallic and covalent components of the total energy of an interatomic bond. This allows us to consider the proposed common reference frame as a system application of energy approach that is recently being used in material science. Russian materials scientist Dr. Sirotkin from the Kazan University (Russia) laid the foundation^10,11^ for the possibility of creating such an approach. They created the simple adequate method of calculation of metallicity, covalence, and ionicity degrees for homogeneous and heterogeneous interatomic bonds. One of the authors of the paper has perfected^12^ this method as applied to steels and alloys. In general, for different interatomic bonds of atoms of the alloy matrix to alloying elements and impurities, the degrees of metallicity, covalence, and ionicity of the bonds are determined by solving the following equations^12^:

1 $${C}_{kPol}=\exp (\,-\,0,\,18\varDelta {\chi }^{2}),$$

2 $${C}_{iPol}=1-{C}_{kPol},$$

3 $$\Delta \chi ={k}_{o}{\chi }_{mat}-{\chi }_{el},$$

4 $${\chi }_{mean}=\frac{{k}_{o}{\chi }_{mat}+{\chi }_{el}}{2},$$

5 $${C}_{kred}=0,253{\chi }_{mean},$$

6 $${C}_{mred}=1-{C}_{\kappa Pol,}$$

7 $${C}_{mi}=\frac{{C}_{mred}}{1+{C}_{iPol}},$$

8 $${C}_{{k}_{i}}=\frac{{C}_{kred}}{1+{C}_{iPol}},$$

9 $${C}_{{i}_{i}}=\frac{{C}_{ired}}{1+{C}_{iPol}},$$

where ${C}_{kPol}$ and ${C}_{iPol}$ are degrees of covalence and ionicity of the Pauling’s formula; Δχ is a difference of the electronegativities of the elements forming the bond; χ_*mean*_ is the mean average value of the electronegativity of the elements forming the bond; χ_*mat*_ is the electronegativity of the atom of the alloy matrix; χ_*el*_ is the electronegativity of alloying element (or impurity), also it is the electronegativity of the matrix atom in the consideration of inter-atomic bonding atoms of the matrix in steel; *С*_*k red*_ and *С*_*m red*_ are the present degrees of covalence and metallicity; *k*_*o*_ is the coefficient equal to the inverse number of nearest neighbors for atoms of alloying elements or impurities forming solid solutions, or it is equal to the inverse of the index by the alloy matrix atom, *A*, in the chemical formula for chemical compounds between *A* and other atoms (in the chemical formula, the index of these atoms should be chosen as equal to one); ${C}_{{m}_{i}}$, ${C}_{{k}_{i}}$ and ${C}_{io{n}_{i}}$ are degrees of metallicity, covalence and ionicity of interatomic bond where *i* is the index of the bond.

The *k*_*o*_ coefficient in Eqs. (3) and (4) is used to account for the chemical bonds order of the alloy matrix atom. If ${k}_{o}=1$, then the system of Eqs. (1–9) is reduced to the system of equations proposed by Sirotkin^10,11^.

For homonuclear bonds, it is generally thought that the ion component of the bond is absent^13^. Therefore, for bonds between the matrix atoms, the degrees of covalence and metallicity of these bonds can be found by Eqs. (1–7) and Eq. (10)^12^:

10 $${C}_{{k}_{i}}=1-{C}_{{m}_{i}}.$$

Because in alloys atoms of alloying elements are substituted into solid solutions, which are unifiedly in the center of the first coordination sphere, (if there is no chemical heterogeneity) surrounded by atoms of the matrix, the apparent cause of a significant local shift of the electron density to a more electronegative element is absent. In this case, we can neglect the ionic component of the heteronuclear bond for bonds between matrix atom to a replacement atom in an alloy. Then the degrees of metallicity and covalence is determined according to the Eqs. (1–7) and (10) ^12^.

As shown by the example of a number of steels and alloys^12^, ${C}_{{m}_{i}}$ and ${C}_{{k}_{i}}$, calculated according to Eqs. (1–7, 10) it characterizes the average statistical parameters of resonating (according to the theory of Pauling^14^) interatomic bonds in the solid solution. For the case of the instantaneous limit state of the fluctuating interatomic bond in the solid solution, the ${C}_{{m}_{i}}$ and ${C}_{{k}_{i}}$ are determined by Eqs. (1–8) ^12^ when the interatomic bond with the metal and covalent components have an instantaneous ionic component.

We will consider nonindividual interatomic bonds but in the future, as a whole, the entire population of interatomic bonds in all structural components of the alloy will be considered. As characteristics of this population of bonds we will use generalized degrees of metallicity and covalence. Considering that ${C}_{{m}_{i}}$ and ${C}_{{k}_{i}}$ of any *i*-th interatomic bond characterizes the energy change of the metallic and covalent components of the bond in its total energy, generalized degrees of metallicity and covalence have a physical meaning on the proportion of energy of all metallic and covalent bonds in the total energy of chemical bonds in all phases of the alloy, respectively.

In this article, we consider the proposed approach in accordance with one of the principles of the cognitive science^15^ “bottom-up”, that is, we analyze specific examples with their subsequent generalization.

## Results and Discussion

### Correlation between the generalized covalence degree of bond atoms of iron to carbon and hardness of microstructures of eutectoid steel

**Figure 1:**
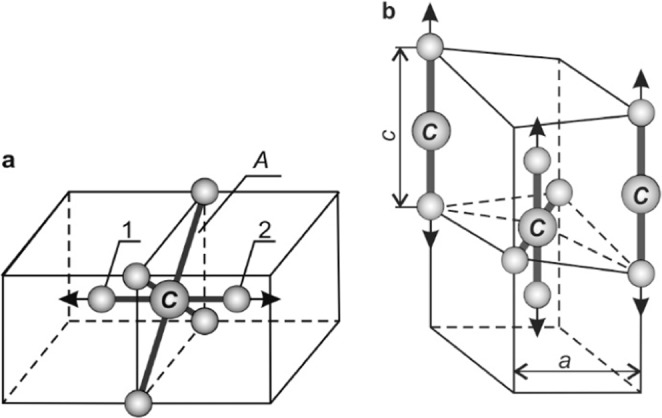
(**a**) The small cluster is Fe_6_C in the bcc-Fe, and **(b**) the possible arrangement of Fe_2_C and Fe_4_C small clusters in the formation of tetragonal distortion of the martensite^8^ crystal lattice. 1, 2 are displaced *Fe* atoms.

In the crystal lattice of α-*Fe*, the placement of carbon atom is energetically most profitable in octahedral pore, which is located in the plane *A*, (see Fig. 1a). In doing so, the atoms *Fe* 1 and 2 are displaced in the direction with a lower packing density of atoms. As a result, the atom *C* will be equidistant about the six nearest *Fe* atoms. We consider this configuration of iron and carbon atoms as the *F*e_6_*C* small cluster consisting of 7 atoms.

In the ferrite and austenite, carbon atoms experience significant repulsion^16^, resulting in their uniform distribution in the solid solution. The repulsion does not rule out the formation of stable small clusters of carbon atoms in ferrite, as long as the distance between pairs of carbon atoms is significant^16^. Besides the *Fe*_6_*C* clusters that we have considered, carbon atoms located in the octahedral interstices in the middle of the edges of body centered cubic ferrite are also able to form clusters^16^; i.e. these are small clusters of *Fe*_2_*C* consisting of three atoms. The formation of these small clusters does not violate the equal distribution of carbon atoms in the solid solution^16^.

According to the carbon atom maps^17^, the carbon atoms fairly evenly distributed in the perlite ferrite. This means that in perlitic, sorbitic and troostitic ferrite, carbon atoms can form small clusters of *Fe*_6_*C* and *Fe*_2_*C* just as it is in ferrite also. In the example of siliceous steels, carbon is mainly evenly distributed in the matrix of bainitic ferrite and its concentration is estimated at 7–16 wt. % C^18^, which corresponds to the average content of 0.7–1.7 carbon atoms per one unit cell of *α*-iron^18^. This corresponds to the condition of a significant distance between pairs of carbon atoms^16^ and means that in the matrix of bainitic ferrite, carbon atoms can form small clusters of *Fe*_6_*C* and *Fe*_2_*C*. In addition, in bainitic ferrite, the carbon atom maps show the existence of aggregate of carbon atoms with a content of 17–26 wt. % C, which corresponds to clusters of *Fe*_4_*C* with 20 wt. % C^19^. Carbon atoms contained in martensite can form small clusters of *Fe*_6_*C*^20^, *Fe*_2_*C*^21^, and *Fe*_4_*C*^20^. Thus, in the perlitic, sorbitic, and troostitic ferrite we consider the availability of small clusters of *Fe*_6_*C* and *Fe*_2_*C*, by also taking into account, we consider the existence of small clusters of *Fe*_6_*C*, *Fe*_2_*C*, and *Fe*_4_*C* in bainitic ferrite and martensite.

One of the possible variants of the spatial location of small clusters of *Fe*_2_*C* and *Fe*_4_*C* is shown in Fig. 1b in the formation of tetragonal distortion of the martensite crystal lattice

The ${C}_{{k}_{F{e}_{2}C}}$, ${C}_{{k}_{F{e}_{4}C}}$, ${C}_{{k}_{F{e}_{6}C}}$ and ${C}_{{k}_{F{e}_{3}C}}$ covalence degrees of the interatomic bond between the iron atom and the carbon atom in *Fe*_2_*С, Fe*_4_*С, Fe*_6_*С* clusters and *Fe*_3_*С* cementite have the values^8^: 0.3141, 0.2438, 0.224 and 0.2654, respectively, calculated according to the Eqs. (1–8) based on the values of the indices of atoms in the chemical formula. It follows that, for example, for *Fe*_2_*C* as a small cluster, cluster or carbide (η-*Fe*_2_*C* or ε-*Fe*_2_*C*), ${C}_{{k}_{F{e}_{2}C}}$ will have the same value. This is a drawback of model (1)–(8), but, as a first approximation, this disadvantage is acceptable. This can be explained in the following example. During aging of martensite at temperatures from 370 K to 470 K, carbide η-*Fe*_2_*C* grows from nucleuses formed in parts of the clustering of carbon^22,23^. The carbide lattices of η-*Fe*_2_*C*, ε-*Fe*_2_*C* and martensite are well combined^24^, consequently, the distances between the iron and carbon atoms in the *Fe*_2_*C* clusters, martensite (in parts of the clustering of carbon), and the carbides of η-*Fe*_2_*C*, ε-*Fe*_2_*C* are approximately the same. In the calculation of the degree of covalence of the interatomic bond^25^ from the quantum mechanical positions, this means that ${C}_{{k}_{F{e}_{2}C}}^{{\rm{cluster}}}\approx {C}_{{k}_{F{e}_{2}C}}^{\eta -\text{carbide}}\approx {C}_{{k}_{F{e}_{2}C}}^{\varepsilon -\text{carbide}}$, which confirms the admissibility of the use of Eqs. (1–8) in the approach under consideration.

In a common cause, for pearlitic, sorbitic, troostitic or bainitic ferrite and ferrite, the generalized covalence degree ${C}_{k}^{F}$ to all *Fe* – *C* bonds in solid solution can be calculated as follows:

11 $${C}_{k}^{F}={n}_{F{e}_{6}C}{C}_{{k}_{F{e}_{6}C}}+{n}_{F{e}_{4}C}{C}_{{k}_{F{e}_{4}C}}+{n}_{F{e}_{2}C}{C}_{{k}_{F{e}_{2}C}},$$

where ${n}_{F{e}_{6}C}$, ${n}_{F{e}_{4}C}$ (for pearlitic, sorbitic, troostitic ferrite and ferrite ${n}_{F{e}_{4}C}=0$) and ${n}_{F{e}_{2}C}$ are proportions of the total number of carbon atoms dissolved in α-*Fe* forming small clusters of *Fe*_6_*С*, *Fe*_4_*С* and *Fe*_2_*С*, respectively.

For perlite, sorbite, troostite and bainite, generalized covalence degree ${C}_{k}^{{g}_{Fe-C}}$ generally characterizing all *Fe* – *C* bonds in ferrite and cementite of these microstructures is determined, taking into cognizance the mass fractions by equation:

12 $${C}_{k}^{{g}_{Fe-C}}={m}_{F}{C}_{k}^{F}+{m}_{cem}{C}_{{k}_{3}}^{Fe-C},$$

where *m*_*F*_ and *m*_*cem*_ are the mass fractions of ferrite and cementite, respectively. The values of *m*_*F*_ and *m*_*cem*_ to the microstructures under consideration calculated on the basis of the law of conservation of mass are as follows in eutectoid carbon steel^8^:

- ${m}_{F}=0,\,881$ and ${m}_{cem}=0,\,119$ for pearlite, sorbite, and troostite when the concentration of carbon in the ferrite of these structural components is 0.008 wt.%^26^;
- ${m}_{F}=0,\,894$ and ${m}_{cem}=0,\,106$ for upper bainite when the content of carbon in the ferrite of upper bainite is 0.1 wt.%^27^;
- ${m}_{F}=0,\,902$ and ${m}_{cem}=0,\,098$ for lower bainite when the carbon concentration in the ferrite of lower bainite is about 0.16 wt.%^27^.

Table 1 presents the generalized covalence degrees ${C}_{k}^{{g}_{Fe-C}}$ for a set of *Fe–C* bonds in possible microstructures of the eutectoid steel, the ferrite, cementite; it also presents the hardness of these structural components and assessments of ${n}_{F{e}_{2}C}$ ${n}_{F{e}_{4}C}$ and ${n}_{F{e}_{6}C}$.

**Table 1:** The generalized covalence degrees ${C}_{k}^{{g}_{Fe-C}}$ of all *Fe–C* bond in the ferrite, cementite, the different possible types of structural components of the eutectoid steel, the hardness of these components and ${n}_{F{e}_{2}C}$ ${n}_{F{e}_{4}C}$, ${n}_{F{e}_{6}C}$.

Structural component	HB, MPa (HRC or НВ, kg/mm^2^)^28^	${{\bf{C}}}_{{\bf{k}}}^{{{\bf{g}}}_{{\bf{F}}{\bf{e}}-{\bf{C}}}}$	${n}_{F{e}_{2}C}$, ${n}_{F{e}_{6}C}$, ${n}_{F{e}_{4}C}$
Ferrite	588 (HB ≈ 60)	0,224	$$\begin{array}{c}{n}_{F{e}_{2}C}\approx 0;\,{n}_{F{e}_{6}C}\approx 1;\\ {n}_{F{e}_{4}C}=0\end{array}$$
Perlite	2000 (HRC ≈ 15)	0,23153 (12)	$$\begin{array}{c}{n}_{F{e}_{2}C}=0,033;\,{n}_{F{e}_{6}C}=0,967;\\ {n}_{F{e}_{4}C}=0\end{array}$$
Sorbite	2800 (HRC ≈ 30)	0,23566 (12)	$$\begin{array}{c}{n}_{F{e}_{2}C}=0,085;\,{n}_{F{e}_{6}C}=0,915;\\ {n}_{F{e}_{4}C}=0\end{array}$$
Troostite	3640 (HRC ≈ 40)	0,24042 (12)	$$\begin{array}{c}{n}_{F{e}_{2}C}=0,145;\,{n}_{F{e}_{6}C}=0,855;\\ {n}_{F{e}_{4}C}=0\end{array}$$
Upper bainite	4125 (HRC ≈ 45)	0,24290 (12)	$$\begin{array}{c}{n}_{F{e}_{2}C}=0,159;\,{n}_{F{e}_{6}C}=0,745;\\ {n}_{F{e}_{4}C}=0,096\end{array}$$
Lower bainite	5075 (HRC ≈ 55)	0,24822 (12)	$$\begin{array}{c}{n}_{F{e}_{2}C}=0,227;\,{v}_{F{e}_{6}C}=0,667;\\ {n}_{F{e}_{4}C}=0,096\end{array}$$
Martensite	7350	0,26 (17)	$$\begin{array}{c}{n}_{F{e}_{2}C}\,{\rm{by}}\,(14);\,{n}_{F{e}_{4}C}\,{\rm{by}}\,(15);\\ {n}_{F{e}_{6}C}\,{\rm{by}}\,(16)\,{\rm{to}}\,C=0,8 \% \end{array}$$
Cementite	8330 (HB ≈ 850)	0,2654	—

The values of ${n}_{F{e}_{2}C}$ in Table 1 have been obtained based on the assumption of the determinative and the proportional contribution of small clusters of *Fe*_2_*C* in the hardening of the pearlitic, sorbitic, troostitic and bainitic ferrite.

**Figure 2:**
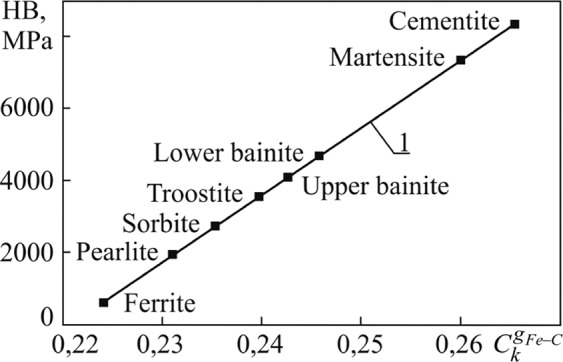
Hardness of ferrite, cementite and various types of possible microstructures (perlite, sorbite, troostite, upper bainite, lower bainite, and martensite) in eutectoid steel, depending on the generalized covalence degree of *Fe-C* bonds^8^. 1 is a regression equation.

The data given in Table 1 are presented in Fig. 2.

The generalized covalence degree ${C}_{k}^{{g}_{Fe-C}}$ of *Fe – C* interatomic bonds equals ${C}_{{k}_{F{e}_{6}C}}$ for ferrite and ${C}_{{k}_{F{e}_{3}C}}$ for cementite in Fig. 2. The parameter ${C}_{k}^{{g}_{Fe-C}}$ for the perlite, sorbite, troostite, lower and upper bainite is calculated by Eq. (12). This parameter for the martensite is determined by Eq. (17). Figure 2 illustrates that the hardness of possible microstructures in the eutectoid steel (perlite, sorbite, troostite, upper bainite, lower bainite, and martensite) and also the hardness of ferrite and cementite correlates to ${C}_{k}^{{g}_{Fe-C}}$. In the approach, we are considering that the generally characterizes ${C}_{k}^{{g}_{Fe-C}}$ the set of *Fe – C* bonds in the microstructure and this parameter has physical implication on the share of energy *E*_*k*_ of all the covalent components of *Fe–C* bonds in the total energy *E*_*g*_ of interatomic bonds of iron and carbon atoms in all elements of this microstructure; ${C}_{k}^{{g}_{Fe-C}}={E}_{k}/{E}_{g}$.

As shown in Fig. 2, the ${C}_{k}^{{g}_{Fe-C}}$ grows when the hardness increases, in so doing, the *E*_*k*_ is also growing. This corresponds to the known understanding that the hardness of materials increases with increase in the number of covalent bonds per unit volume^29^. On the other hand, the hardness of different microstructures of eutectoid steel can be formed by various hardening mechanisms, such as the solid-solution hardening, grain boundary hardening, dispersion hardening, martensitic transformation, etc. However, if we will look at these mechanisms of hardening through the prism of the atomic level, we will discover that the result of their action is only in the form of relative displacement or movement of a part of Fe and C atoms. In particular, the parameters ${n}_{F{e}_{2}C}$, ${n}_{F{e}_{4}C}$ and ${n}_{F{e}_{6}C}$ take into account such displacement of atoms (see Table 1). These parameters define ${C}_{k}^{{g}_{Fe-C}}$ according to Eq. (11). Therefore we consider ${C}_{k}^{{g}_{Fe-C}}$ exclusively as the present factor related to the atomic level, which reflects in its values the result of strengthening through correlation with hardness. Thus, Fig. 2 illustrates that the generalized covalence degree of *Fe – C* interatomic bonds, which is the parameter of the atomic hierarchical level of a metal structure, can be reflected through correlation with the hardness of microstructures, the result of mechanisms that strengthens these microstructures.

Equation 1 in Fig. 2 is $HB=-\,41301,\,08+187004,\,83{C}_{k}^{{g}_{Fe-C}}$ with *R* = 0,999. This equation in units of hardness HRC (at HRC ≥ 12) with *R* = 0,997 have the form (conversion between different hardness units was carried out according to ISO 18265):

13 $$HRC=-\,17040,\,87+193296,\,32{C}_{k}^{{g}_{Fe-C}}-730579,\,208{({C}_{k}^{{g}_{Fe-C}})}^{2}+923819,\,288{({C}_{k}^{{g}_{Fe-C}})}^{3}.$$

Martensitic transformation has a non-diffusion character and is carried out by the corporate movement of atoms. The carbon atoms share ${n}_{F{e}_{2}C}(C)$ in octahedral pores in the center of edges. In the formation of *Fe*_2_*C*, small clusters (see Fig. 1b) can be considered proportional to the known angular coefficient of the concentration dependence of the crystal lattice period of martensite $\alpha =0,\,118$^27,30^ in cases where the processes of carbon atoms redistribution in the interstices ended. That is,

14 $${n}_{F{e}_{2}C}(C)={b}_{l}+0,\,118C$$

where *C* is the mass concentration of carbon in steel; *b*_*l*_ is the coefficient; $l=1,\,2$.

To determine the coefficients *b*_1_ and *b*_2_ we believe that in a steel at the initial stage of formation of tetragonal distortion of the α-*Fe* lattice:

- The probability of placing an atom *C* in any of the 18^16,21^ octahedral pores of the elementary crystal lattice α-*Fe* is *P*_*p.o*._; ${P}_{p.o.}=1/18$;
- The probability of a joint occurrence of two independent events of placement of *C* atoms in any of the 12^16,21^ octahedral pores in the center of edges of the unit cell, the α-*Fe* lattice to the formation of the two clusters of *Fe*_2_*C* is *P*_*pair*_; ${P}_{pair}=1/(12\cdot 12)$.

In Eq. (14)${b}_{1}={P}_{p.o.}+{P}_{pair}-{P}_{p.o.}{P}_{pair}=0,\,0621$ if the joint appearance of all the above cases and at $C < 0,16\, \%$.

At $C\ge 0,16\, \%$ ${n}_{F{e}_{2}C}(C)$ receives the value of probability of two joint events of *C* atoms placement on edges to the formation of small clusters *Fe*_2_*C* and in this case: ${b}_{2}={P}_{p.ad.}+{P}_{p.ad.}-{P}_{p.ad.}{P}_{p.ad.}=0,\,1597$ where *P*_*p.ad*._ is the probability of occupying any position from 12^16,21^ octahedral interstices in the center of edges in a unit cell of α-*Fe*.

The placement of carbon atoms in octahedral voids with the formation of the small clusters *Fe*_4_*C* is a random process and the logarithmic normal distribution is the most suitable for its description. In this case, the share of carbon atoms in the formation of the *Fe*_4_*C* small clusters is determined by the equation:

15 $${n}_{F{e}_{4}C}\left(C\right)=\frac{1}{C{\sigma }_{l}\sqrt{2\pi }}{\exp }\left[-\frac{{\left(\mathrm{ln},C\right)}^{2}}{2{\sigma }_{l}^{2}}\right],$$

where σ_*l*_ is the standard deviation. In Eq. (15), the σ_*l*_ values that provide the best fit between the estimated hardness of martensite and the experimental data are ${\sigma }_{1}=\,\mathrm{ln}\,2,96$ (at$C < 0,\,16\, \%$) and ${\sigma }_{2}=\,\mathrm{ln}\,2$ (at $C\ge 0,\,16 \%$).

The share of carbon atoms that form the small clusters of *Fe*_6_*C* is given as:

16 $${n}_{F{e}_{6}C}(C)=1-[{Q}_{F{e}_{2}C}(C)+{Q}_{F{e}_{4}C}(C)].$$

According to the additivity rule, the generalized covalence degree of all the *Fe–C* bonds in martensite ${C}_{k}^{{g}_{Fe-C}}(C)$ is determined by the equation:

17 $${C}_{k}^{{g}_{Fe-C}}(C)={C}_{{k}_{F{e}_{6}C}}{n}_{F{e}_{6}C}(C)+{C}_{{k}_{F{e}_{2}C}}{n}_{F{e}_{2}C}(C)+{C}_{{k}_{F{e}_{4}C}}{n}_{F{e}_{4}C}(C).$$

**Figure 3:**
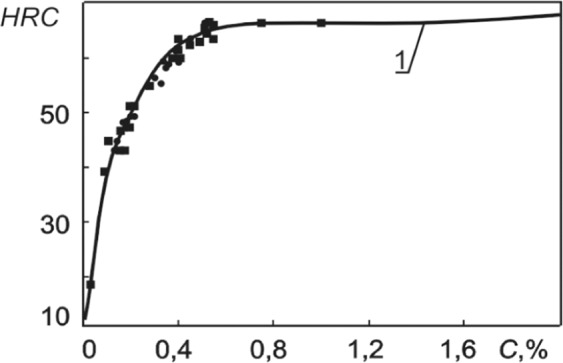
The hardness of martensite depending on the carbon mass content^8^. 1 is the solution of Eqs. (13–17). Experimental data^26^: ■ is carbon steel; ● is steel alloy.

The solution of Eqs. (13–17) is given in Fig. 3.

The mathematical functional dependence 1 (the solution of Eqs. (13–17)) of the hardness of martensite on the mass concentration of carbon shown in Fig. 3 is received for the first time. It corresponds to the known experimental data $R=0,\,975$. The result, on the one hand, confirms the point made earlier that ${C}_{k}^{{g}_{Fe-C}}$ is capable of reflecting, through its correlation with the hardness of the microstructure, the result of the action of various hardening mechanisms responsible for the hardness of this microstructure; on the other hand, this result demonstrates the fruitfulness of the developed approach.

Thus, here we have shown that the characteristic of the atomic hierarchical level of the metal structure (a generalized covalence degree ${C}_{k}^{{g}_{Fe-C}}$ of interatomic *Fe–C* bonds) reflects the existence of nanostructured formations (i.e, various small clusters of *Fe*_2_*С, Fe*_4_*С* or *Fe*_6_*С*), allows to identify the type of microstructure (i.e ferrite, pearlite, sorbite, troostite, upper bainite, lower bainite, martensite, and cementite) through correlation with the hardness of these microstructures. Considering this on the other hand, the ${C}_{k}^{{g}_{Fe-C}}$ reflects at the atomic level the result of the various hardening mechanisms responsible for the hardness of these microstructures.

The following section will consider the generalized degrees of metallicity and covalence not for the set of *Fe–C* bonds but for the set of absolutely all interatomic bonds in all phases in alloy.

### Correlation of the generalized degrees of metallicity and covalence for the set of all interatomic bonds in all phases of alloy to the solid-solution hardening of a number of steels and alloys

The solid-solution hardening is realized due to the implementation of a number of the following physical processes.

The doping elements create a field of elastic distortion of the crystal lattice, causing the retardation of dislocations, changing the energy of stacking fault, influencing the magnitude of Payers-Nabara’s forces, and also increasing the friction force during the motion of an edge dislocation, which makes it difficult for cross sliding when traversing the dissolved atom^31^.

As established by Cottrell, Hunter and Nabarro, the electrical interaction of dislocations and a solute atom inhibits dislo cation movement. However, the energy of this interaction is several times less than the energy of their elastic interaction^32^.

Obstacles to dislocations occur due to complication in the intragranular structure and the formation of surface defects such as antiphase domain boundaries and the domains boundary. Foreign atoms forming the Cottrell’s, Snuka’s or Suzuki’s atmospheres impede the movement of dislocations. The interaction of elements that are dissolved in the crystal lattice of the matrix also affects the degree of deformation hardening^31^.

In accordance with the physical representations that develops in the theories of Mott’s & Nabarro’s^33^, Fleischer’s^34^, Friedel’s^35^, and Labouche’s^36^, the mechanisms of solid-solution hardening is based on the elastic interaction conception of alloying elements to dislocations. The implication is that the replacement of the matrix atom by the atom of the alloying element will cause the displacement of neighbouring atoms. This causes the emergence of elastic deformation and associated stresses in the matrix^37^. However; the atomic radius is defined as half the interatomic distance and depends on the state of the interatomic bond^38^. Consequently, the changes in the characteristics of the state of the interatomic bond, including degrees of metallicity and covalence, would be correlated to solid-solution hardening with the appearance of elastic deformation in the matrix. Such a correlation for a number of steels and alloys is determined by following regression equations^7,12^:

18 $${\sigma }_{y}={a}_{{\sigma }_{y}}+{b}_{{\sigma }_{y}}{C}_{m}^{ss}+{d}_{{\sigma }_{y}}{C}_{k}^{is},$$

19 $${\sigma }_{u}={a}_{{\sigma }_{u}}+{b}_{{\sigma }_{u}}{C}_{m}^{ss}+{d}_{{\sigma }_{u}}{C}_{k}^{is},$$

20 $$\delta ={a}_{\delta }+{b}_{\delta }{C}_{m}^{ss}+{d}_{\delta }{C}_{k}^{is},$$

21 $$\psi ={a}_{\psi }+{b}_{\psi }{C}_{m}^{ss}+{d}_{\psi }{C}_{k}^{is},$$

where σ_*y*_ is yield strength; σ_*u*_ is the ultimate tensile strength; δ is the elongation; ψ is the relative contraction; ${a}_{{\sigma }_{y}}$, ${a}_{{\sigma }_{u}}$, ${a}_{\delta }$, ${a}_{\psi }$, ${b}_{{\sigma }_{y}}$, ${b}_{{\sigma }_{u}}$, ${b}_{\delta }$, ${b}_{\psi }$, ${d}_{{\sigma }_{y}}$, ${d}_{{\sigma }_{u}}$, ${d}_{\delta }$, ${d}_{\psi }$ are coefficients that are determined by regression analysis; ${C}_{m}^{ss}$ is a generalized degree of metallicity of the entire set of interatomic bonds in the substitution solution; ${C}_{k}^{is}$ is a generalized degree of covalence, which generally characterizes all the bonds between atoms in the interstitial solid solution. Therefore we have^7,12^:

22 $${C}_{m}^{ss}=\sum _{j}{C}_{{m}_{j}}\,{X}_{j},$$

23 $${C}_{k}^{is}=\sum _{i}{C}_{{k}_{l}}\,{X}_{l},$$

where *i*, *j* are the indexes of chemical bonds in the substitution solid solution and interstitial solid solution, respectively; ${C}_{{m}_{j}}$ is the degree of metallicity of *j*-th interatomic bond; *X*_*j*_ is mole fraction of atom in the substitution solid solution, which is calculated without taking into account interstitial atoms in the chemical composition of steel or alloy; *X*_*i*_ is mole fraction of elements in the interstitial solid solution, which is determined without taking into account the presence of substitutional atoms in the matrix; ${C}_{{k}_{l}}$ is the degree of covalence of *i*-th interatomic bond.

**Figure 4:**
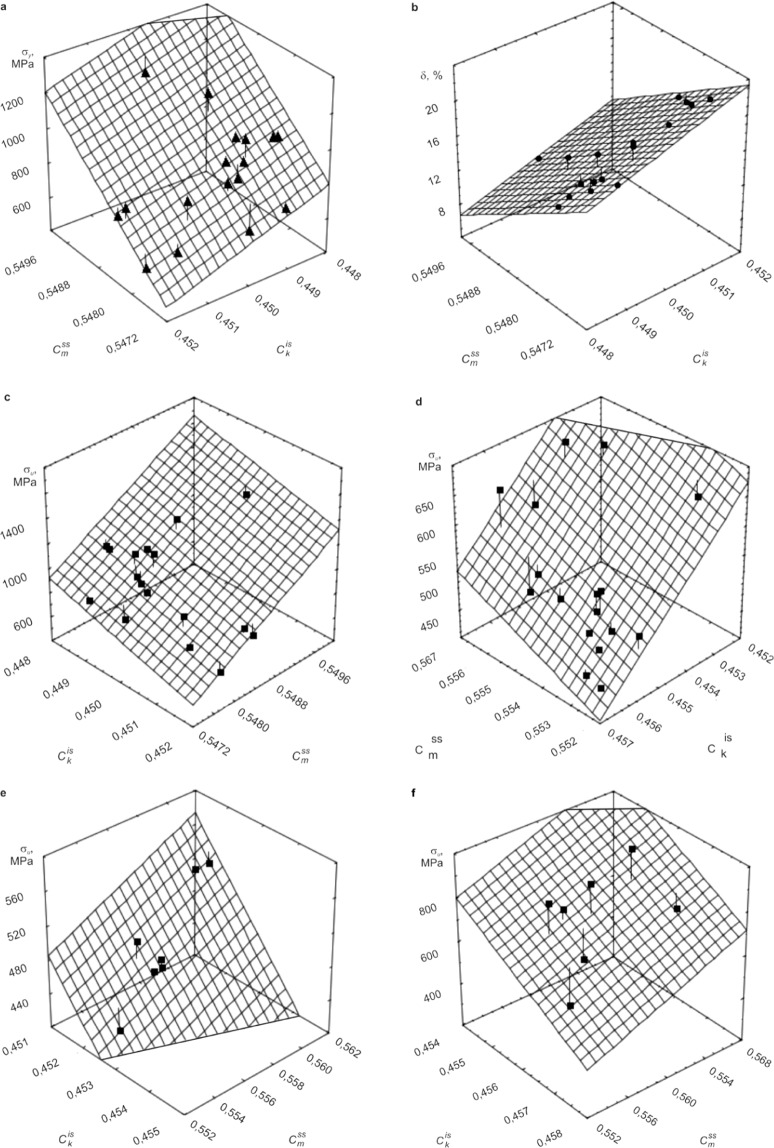
Regression planes of mechanical properties of steels and alloys dependent on generalized degrees of metallicity and covalency in the solid-solution hardening and experimental data.^12^ (**a**) Yield strength of a number of heat-treated low-alloy steels (*R* = 0.89). (**b**) Elongation of a number of heat-treated low alloy steels (*R* = 0.92). (**c**) Ultimate tensile strength of a number of heat-treated low alloy steels (*R* = 0.89). (**d**) Ultimate tensile strength of a number of austenitic stainless steels (*R* = 0.89). (**e**) Ultimate tensile strength of a number of ferritic stainless steels (*R* = 0.95). (**f**) Ultimate tensile strength of a number of the austenitic iron-nickel and nickel alloys (*R* = 0.91).

Graphs that are statistically significant with a confidence probability equal to 0.999 regression equations of the type (18)–(20) for a number of steels and alloys^12^ are given in Fig. 4.

Various mechanisms of hardening additively form the yield strength of steels^39^.

24 $${\sigma }_{y}={\sigma }_{0}+\Delta {\sigma }_{ss}+\Delta {\sigma }_{dis}+\Delta {\sigma }_{ag}+\Delta {\sigma }_{gb},$$

where Δσ_*ss*_, Δσ_*dis*_, Δσ_*ag*_ and Δσ_*g b*_ are the contributions of solid-solution, dislocation, dispersion, and grain boundary hardening mechanisms, respectively; σ_0_ is the Peierls–Nabarro stress.

As can be seen from Eq. (24), the influence of Δσ_*ss*_ on the mechanical properties of steels and alloys can only be identified when the following condition is met^7^:

25 $$\Delta {\sigma }_{dis}+\Delta {\sigma }_{ag}+\Delta {\sigma }_{gb}\approx const.$$

In view of the foregoing, the experimental data and their approximation (see Fig. 4) are considered for the case when condition (25) is met. In doing so, the correlation was obtained between the change in mechanical properties during solid-solution hardening and ${C}_{m}^{ss}$, and ${C}_{k}^{is}$ in representative collecting of samples of the following steels and alloys:

- 17 various low-alloy steels (with 0.2–0.4 wt.% C) subjected to quenching (with full hardenability of samples) and subsequent tempering by which martensite transformed to sorbite (Fig. 4a–c) (Note: In Eq. (23) for the bond Fe – C, we used as ${C}_{kl}$ the value of ${C}_{k}^{{g}_{Fe-C}}$ calculated by Eq. (12));
- 17 different austenitic stainless steels (Cr–Ni and Cr–Mn stainless steels) subjected to austenization (Fig. 4d);
- 7 sheet ferritic stainless steels subjected to annealing (Fig. 4e);
- 7 austenitic iron-nickel and nickel alloys in the form of hot-rolled sheet metal after quenching (Fig. 4f).

Thus, in this section we have shown that the results of the action of the solid-solution hardening are reflected in the values of ${C}_{m}^{ss}$ and ${C}_{k}^{is}$ through their correlation with the mechanical properties of the considered steels and alloys.

### Correlation of the ratio of the generalized degrees of metallicity and covalence for the set of all interatomic bonds in all phases in the alloy to the tensile strength of wrought aluminium alloys

The generalized degrees of metallicity ${C}_{m}^{gAl}$ and covalence ${C}_{k}^{gAl}$ to the totality of all bonds between the matrix atoms and the atoms of substitution are given by the following equations^8^:

26 $${C}_{k}^{gAl}=\sum _{i}{C}_{{k}_{i}}{X}_{i};\,{C}_{m}^{gAl}=\sum _{i}{C}_{{m}_{i}}{X}_{i},$$

where ${C}_{{k}_{i}}$ and ${C}_{{m}_{i}}$ are the degrees of covalence and metallicity of *i*-th interatomic bond, respectively; *X*_*i*_ is the molar fraction of *i*-th dissolved atom involved in the formation of *i*-th chemical bond.

**Figure 5:**
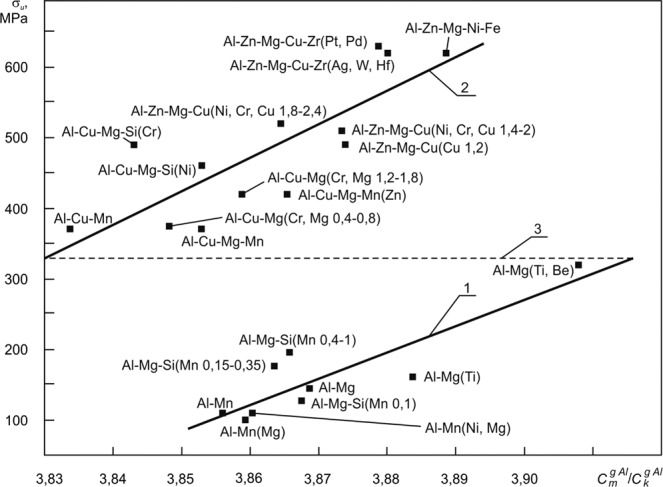
The dependence of the ultimate tensile strength of wrought aluminum alloys of various doping systems on ${C}_{m}^{gAl}/{C}_{k}^{gAl}$ ^8^. The case of the instantaneous limit state of the fluctuating interatomic bond of matrix atoms with substitution atoms is considered, in which the interatomic bonds along with the metallic and covalent components also have an instantaneous ionic component. ▪ are experimental data^40–43^. 1, 2 are regression Eq. 3 is a conditional line of transition from the dependence 1 to the dependence 2.

In Fig. 5 we have shown that ultimate tensile strength^40–43^ of wrought aluminum alloys (both thermally hardened by aging after quenching without polymorphic transformation and thermally non-hardening alloys) of various doping systems correlates with ${C}_{m}^{gAl}/{C}_{k}^{gAl}$.

The wrought non-heat-treatable aluminum alloys (hot-rolled metal) occupy the bottom part, and the wrought heat-treatable aluminum alloys (subjected to aging after quenching without polymorphic transformation) are located in the top part in Fig. 5. All alloys considered were not subjected to strain hardening.

Regression Eq. 1 with *R* = 0.84 relates to wrought non-heat-treatable aluminum alloys, and regression Eq. 2 with *R* = 0.8 relates to wrought heat-treatable aluminum alloys (see Fig. 5).

The main strengthening mechanisms of the alloys under consideration are solid-solution hardening and precipitate hardening, in so doing, the parameter δ_*d*_ increases with increasing temporal resistance in precipitate hardening. This parameter is given by the equation

27 $${\delta }_{d}=|{{\rm{\rho }}}_{d}-{{\rm{\rho }}}_{m}|,$$

where ρ_*d*_ is the density of the strengthening particles of the dispersion phase or clusters (Guinier-Preston zones); ρ_*m*_ is the density of the alloy matrix^8^.

At ${\delta }_{d}\approx 1500\,{\text{kg}/m}^{3}$ an abrupt transition is observed (conditional line 3 in Fig. 5) from the end of the dependence 1 to the beginning of the dependence 2, which can be associated with enhancing the efficiency of hindering of dislocations in the elastic stress field in the neighborhood of particles of the dispersion phase or Guinier-Preston zones at ${\delta }_{d} > 1500\,{\text{kg}/m}^{3}$ ^8^. Also, this indicates that dispersion hardening is the defining strengthening mechanism in the considered wrought heat-treatable aluminum alloys.

Thus, in this section we demonstrated that the result of the action of the dispersion hardening mechanism is reflected by the parameter ${C}_{m}^{gAl}/{C}_{k}^{gAl}$ through the correlation (regression Eq. 2 in Fig. 5) with tensile strength of wrought heat-treatable aluminum alloys (aging after quenching without polymorphic transformation).

### The potentiality for the description of the hardening by means of deformation and grain boundaries in the developed approach

As is known, in the classical approach, the specific conductivity κ of the metal is determined by the equation^44^:

28 $${\rm{\kappa }}=\frac{{N}_{f}{e}^{2}l}{mv},$$

where *N*_*f*_ is the concentration of free electrons; *e* and *m* are the charge and mass of the electron, respectively; *l* is the electron mean free path; *v* is the average speed of the thermal motion of the electron.

In the metal, oscillations of κ occur when dislocations move, in so doing κ does not depend on the dislocation density^45^. Hence, in Eq. (28) $\frac{l}{v}\approx const$ and κ changes proportional to *N*_*f*_. Since the value of *N*_*f*_ is related to the degrees of metallicity ${C}_{mi}$ of interatomic bonds, we can assert that the variations of generalized degree of metallicity are able to reflect the movement of dislocations.

The increase in dislocation density is accompanied by their displacement; including the work sources of Franck-Reed during strain hardening.

The grain-boundary hardening is characterized by the change in motion (braking and stopping) of dislocations in front of the barrier (grain boundary).

It follows from the foregoing that the potential to develop a theoretical description of the hardening by means of a deformation and grain boundaries of the metal exists in the framework of the approach envisaged, if formal methods will be developed that take into account the reflection of the motion of dislocations in variations of the generalized degree of metallicity of interatomic bonds.

### The possibility of practical application of a new approach to the designing of structural alloys at this stage of its development

Technological regimes of manufacturing processes of serial and mass production are constant and stable, virtually. In the circumstances, the action of dislocation, dispersion and grain boundary hardening mechanism gives almost invariable result, that is, the condition (25) is observed. In so doing, the chemical composition of the steel used is the randomly variable factor (it changes randomly since when steel is obtained, not a specific concentration, but the permissible range of concentrations of chemical element is regulated). Consequently, for batch and mass production, the variations of mechanical properties of metal in the finished product are determined by solid-solution hardening. In doing so, in Eqs. (18–21) the independent variables ${C}_{m}^{ss}$ and ${C}_{k}^{is}$ according to Eqs. (22) and (23) can have the same values for different variants of the content of alloying elements and impurities. This is seen as follows. In the case where the condition (25) is true and the coefficients of the regression Eqs. (18–21) are known, we can best adjust the chemical composition technologically during steel melting so that the desired values ${C}_{m}^{ss}$ and ${C}_{k}^{is}$ are set. In this case, according to Eqs. (18–21), the required and stable level of mechanical characteristics through the expense to solid-solution hardening will be provided in parts that will be mass-produced from this steel.

To achieve the desired tensile strength to wrought aluminum alloys, which are thermally hardened by aging after quenching without polymorphic transformation, we can design new doping systems of any complexity. To do so, for example we choose a technologically viable chemical composition (δ_*d*_ must be more than 1500 kg/m^3^) of a new aluminum alloy and optimize it with the use of Eq. (26) so that the parameter ${C}_{m}^{gAl}/{C}_{k}^{gAl}$ has the desired value seen in chart 2 in Fig. 5.

It should be noted that Russian physicists Kazimirov, Smirnov, Balagurov and Natkanets from the Joint Institute for Nuclear Research used a close approach in the implementation of the project: “An integrated method of investigation of promising functional materials by quantum chemistry, neutron scattering and optical spectroscopy”. In this project, when new materials were created, researchers analyzed the atomic structure (local atomic configurations), which “photographed” the consequences of the interaction of different elements of the structure of the material. This “picture” was used to analyze the results whether the properties of the material have been changed purposefully in the right way. This approach has been recognized as universal and fruitful in JINR^46^.

## Summary

In this paper we have considered a number of specific examples where the generalized degrees of metallicity and covalence, which generally characterizes the entire set of interatomic bonds in all phases of the alloy, are reflected through the correlation with the following mechanical properties:

1. It is the result of solid-solution hardening and precipitate hardening (The results of strain hardening and grain boundary hardening could potentially be taken into account);
2. It is the strengthening result by quenching (we obtained a mathematical functional dependence between the hardness of martensite and the carbon concentration in steel when the generalized degree of covalence of the bond between the atoms of iron and carbon was analyzed);
3. It is the characteristics of structure, namely, the form of nanostructure (various small-scale clusters of carbon with iron) and the type of microstructure (ferrite, perlite, sorbitol, troostite, lower bainite, upper bainite, martensite, and cementite) in steel.

It appears from the foregoing that the parameters of the atomic level structure of the metal (the generalized degrees of metallicity and covalence) could also reflect in their values, through the correlation with mechanical properties of the metal, the result of the influence of factors (chemical composition and distribution of elements, structure and phase composition, effect of defects, technological processes involved from the smelting of the alloy to the manufacture of the product, etc.) that determine these properties of metal in the finished product. That is, the atomic level is capable of fulfilling the role of common frame of reference in which we can compare the action and interaction of all these factors, and also the effect of various strengthening mechanisms. Apparently, the combined use of the theoretical and experimental (study of local atomic configurations) approaches considered has a good prospect for the designing of new structural materials.
